# Fast positioning for X-ray scanning microscopy by a combined motion of sample and beam-defining optics

**DOI:** 10.1107/S160057751801785X

**Published:** 2019-01-25

**Authors:** Michal Odstrcil, Maxime Lebugle, Thierry Lachat, Jörg Raabe, Mirko Holler

**Affiliations:** a Swiss Light Source, Paul Scherrer Institute, Villigen 5232, Switzerland; b Laboratory for Micro and Nanotechnology, Paul Scherrer Institute, Villigen 5232, Switzerland

**Keywords:** ptychography, X-ray imaging, Fresnel zone plate

## Abstract

Scanning X-ray microscopy such as X-ray ptychography requires accurate and fast positioning of samples in the X-ray beam. Sample stages often have a high mobile mass as they may carry additional mechanics or mirrors for position measurements. The high mobile mass of a piezo stage can introduce vibrations in the setup that will lead to imaging quality deterioration. Sample stages also require a large travel range which results in a slow positioning step response and thus high positioning overhead. Moving lightweight X-ray optics, such as focusing Fresnel zone plates, instead of the sample can improve the situation but it may lead to undesired variations in the illumination probe which may result in reconstruction artifacts. This paper presents a combined approach in which a slow sample stage mechanism covers the long distance range for a large field of view, and a light-weight optics scanner with a small travel range creates a superimposed motion to achieve a fast step response. The step response in the ptychographic tomography instrument used was thereby improved by an order of magnitude, allowing for efficient measurement without loss of imaging quality.

## Abstract 2

A novel approach for fast ptychography scans over an extended field of view by means of simultaneous Fresnel zone plate and sample motion is presented.

## 1. Introduction   

In scanning X-ray microscopy, either the sample is shifted through a beam (Shapiro *et al.*, 2014 ▸; Menzel *et al.*, 2010 ▸) or the X-ray optics are shifted with respect to the sample (Takeichi *et al.*, 2016 ▸; Medjoubi *et al.*, 2013 ▸; Kilcoyne *et al.*, 2003 ▸, 2010 ▸; Klug *et al.*, 2018 ▸) and different types of signals are recorded, such as the total or diffraction intensity of transmitted light or an X-ray fluorescence signal emitted by the sample (Kaulich *et al.*, 2011 ▸; Chen *et al.*, 2014 ▸). In contrast to electron microscopy, the manipulation of the photon beam itself is rather difficult and usually requires physically moving the sample or the optics in the steady beam. Typically, data acquisition is either in the fly scan mode (Klug *et al.*, 2018 ▸; Medjoubi *et al.*, 2013 ▸; Menzel *et al.*, 2010 ▸), when the sample or optics is continuously moved through the beam, or the scanning is pointwise (Celestre *et al.*, 2017 ▸; Shapiro *et al.*, 2014 ▸), when the motion is paused during signal acquisition in order to avoid a spatial smearing and reduction of the imaging quality. It is therefore desirable to minimize the overhead caused by mechanical movements and to perform such movements as fast as possible.

In our specific case, we illuminate a sample with coherent X-rays with a photon energy of a few keV and record coherent far-field diffraction patterns at overlapping sample positions. Complex-valued transmission and illumination functions are reconstructed using iterative algorithms, which solves the phase problem (Thibault *et al.*, 2008 ▸; Rodenburg *et al.*, 2007 ▸). This method is called X-ray ptychography and the imaging resolution that can be obtained is neither limited by the step size in the scan nor by the illumination probe size used in the experiment. Instead, resolution is determined by the positioning accuracy of the sample in the beam and the maximum collected scattering angle with sufficient signal-to-noise ratio. The beam size that can be used in the experiment is determined by the X-ray energy and pixel size of the detector. In our case, this ranges between 2 µm and 10 µm. Therefore, in order to have good spatial overlap in the scan, as required by the reconstruction algorithm, typical step sizes are in the range 0.4 µm to 2 µm. The required total scan range is thereby determined by the dimensions of the sample and in our case it is up to 200 µm. The imaging resolution range we target is sub-10 nm, meaning that the positioning accuracy of the illumination probe on the sample and stability during exposure has to be better than that.

X-ray ptychography can also access 3D information by recording several projections of a sample at different sample orientations and using computed tomography to reconstruct the volume (Guizar-Sicairos *et al.*, 2011 ▸; Dierolf *et al.*, 2010 ▸). In our instrumentation we use diffractive X-ray lenses, so-called Fresnel zone plates (FZPs) (Gorelick *et al.*, 2010 ▸), to define a structured illumination probe on the sample. Due to a high angular stability of the X-ray beam, the position of the illumination probe is mainly defined by the position of the FZP. In order to accurately position a sample in the beam, we have equipped our instruments with dedicated position metrology based on laser interferometry compatible with the rotational degree of freedom required for tomography so that the position of the sample with respect to the FZP can be measured (Holler & Raabe, 2015 ▸). The sample is installed on a 3D piezo stage, which is operated in closed-loop to the interferometer signal. The metrology requires a reference mirror on the sample stage which to a large extent determines the mobile mass. For example, the mobile mass in the tomography instrument described by Holler *et al.* (2012 ▸, 2014 ▸) is 150 g and is installed on a Physikinstrumente P-563 with a 300 µm travel range. The mobile mass combined with the large travel range leads to a low resonance frequency of 115 Hz and thereby to positioning times in the range 50 ms to 80 ms for our typical step sizes. This overhead is comparable with commonly used exposure times for ptychographic experiments at the cSAXS beamline, Paul Scherrer Institut, meaning that the positioning overhead can be more than 50%.

The FZP lens is a small structure patterned using electroplating in PMMA molds on a silicon nitride membrane (Vila-Comamala *et al.*, 2010 ▸). Therefore, it has an extremely low mass of less than 10 mg. A small transversal movement of the lens leads to an effective movement of the illumination probe. This approach is already used in other ptychography instruments (Celestre *et al.*, 2017 ▸; Klug *et al.*, 2018 ▸; Vine *et al.*, 2012 ▸) to move the lightweight lens instead of the sample stage and thus reducing the positioning overhead. However, standard ptychography algorithms are based on the assumption of having a stable illumination probe during the scan. Since the X-ray wavefront illuminating the FZP is, in most hard X-ray beamlines, smooth but not entirely flat, a movement of the FZP lens over a large range leads to undesirable variations of the illumination probe. Additionally an FZP requires an order-sorting aperture (OSA) and a central stop (CS) to block the zero and higher diffraction orders of the lens and a stationary OSA would limit the field of view (Shapiro *et al.*, 2017 ▸). For large range movements, the CS and OSA also have to move synchronously with the FZP (Celestre *et al.*, 2017 ▸).

## 2. Hybrid scanning method   

**Figure 1:**
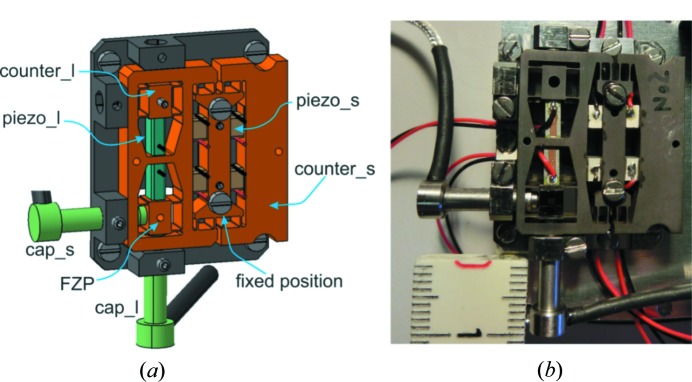
(*a*) Rendering of the FZP scanner (cap_s/l = capacitive sensor short/long axis; counter_s/l = counter mass short/long axis; piezo_s/l = one of the piezo elements short/long axis). (*b*) Photograph of the actual device. The ruler shows 1 cm.

**Figure 2:**
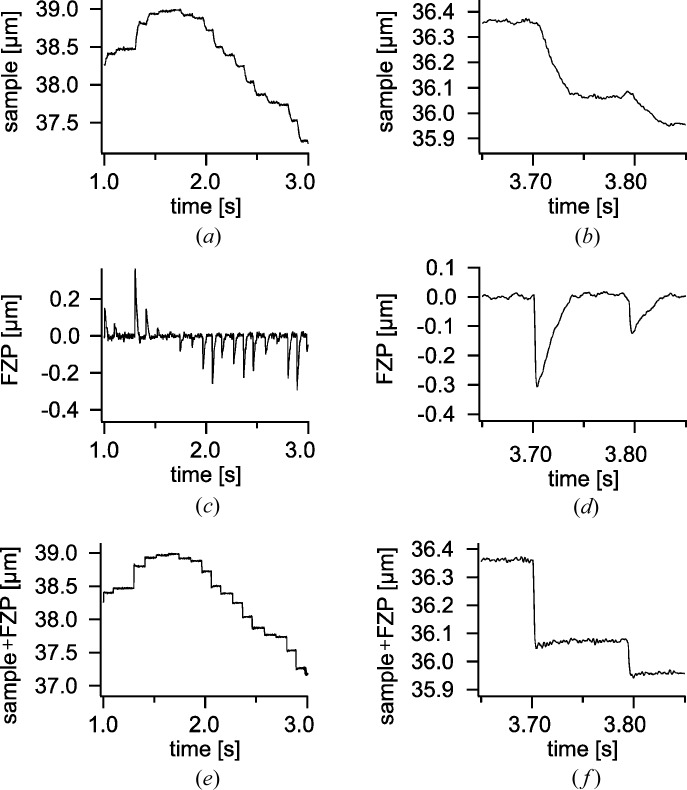
Typical movements of (*a*) and (*b*) the sample stage, (*c*) and (*d*) FZP scanner during a point-by-point scan. Panels (*e*) and (*f*) show the resulting effective motion of the sample relative to the FZP position versus time. The step response of the system can be improved by an order of magnitude and reaches less than 5 ms for an average step size of 0.6 µm. The standard deviation between time marks 3.7 s and 3.8 s in (*f*) is 6.6 nm.

As a solution to these issues we have developed a hybrid approach. A large scanning range is provided by a slow sample stage aided locally by an overlaid fast movement of the FZP covering a small range. To achieve such motion, a custom scanning mechanism for the FZP has been developed and is schematically depicted in Fig. 1 ▸(*a*). It offers a travel range of 3 µm × 5 µm and is based on a flexure structure with outer dimensions of 24 mm × 22 mm × 4 mm. The flexure structure was prepared by wire erosion from a single piece of titanium in order to maximize lifetime at the given tension of the flexures. This structure is driven by small piezo stacks (PCh150/3x3/2 and PSt150/2x3/5, Piezomechanik GmbH, Germany) operated by analog piezo amplifiers (SVR 150, Piezomechanik GmbH, Germany). The scanner uses capacitive sensors as internal position metrology (Lion precision Inc.; C3R-0.5–2.0 probe) that offer a measurement range of 10 µm, such that even though it is an analog metrology the resolution of <1 nm is possible at a 10 kHz bandwidth. Capacitive sensors permit us to keep mobile masses of 0.3 g and 2.2 g in the vertical and horizontal directions, respectively, which would be difficult to achieve for an interferometry feedback that requires additional mirrors attached to the FZP scanner. The compact and stiff design leads to resonance frequencies of a few kHz such that the response of the system is mainly determined by the bandwidth of the piezo amplifier. An open-loop full-range step response of 1 ms is possible, but in closed-loop operation typically 5 ms for 0.6 µm step length are currently achieved. This overhead is comparable with the values that other state-of-the-art ptychography scanners need to reach a 30× shorter step size (Celestre *et al.*, 2017 ▸). Compensation for the sample stage motion leads to a spiky motion trajectory of the fast FZP scanner [see Figs. 2 ▸(*c*) and 2(*d*)] that can excite vibrations in the ptychography scanner and thus deteriorate the reconstruction quality. Therefore, the design of our fast scanner is symmetrical and equipped with counter masses moving in opposite directions such that zero momentum is transferred to the structure holding the scanner. However, later it was experimentally verified that the holding structures in our instrumentation offer sufficient stiffness and such counter-masses are not required due to the low mobile mass of our fast scanner. A photograph of the scanner is shown in Fig. 1 ▸(*b*).

**Figure 3:**
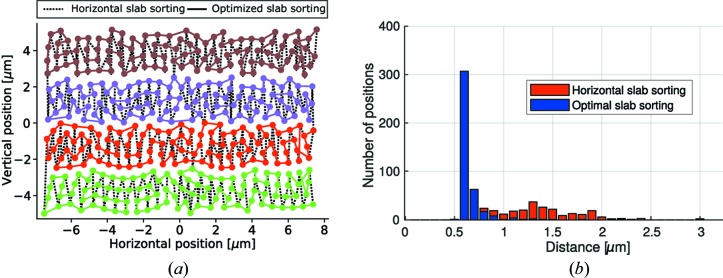
(*a*) The positions of the scan are denoted by dots connected by black dashed lines corresponding to a simple horizontal slab sorting, and full lines are used for the case of the optimal slab sorting with a different color for each 3 µm-wide slab. (*b*) Corresponding histogram showing step length between the subsequent scan positions sorted by both methods. The optimal slab sorting method reorders the positions so that almost all steps can be directly reached by the fast FZP scanner without waiting for the slower sample motion. This leads to a reduced overhead.

The resulting metrology of our instrument is a combined system of laser interferometry measuring the sample position with respect to the holding structure of the FZP scanner and the capacitive signal measuring the effective position of the FZP lens. Fig. 2 ▸ shows the individual position signals of the interferometry corresponding to the sample motion [2(*a*), zoomed 2(*b*)], the capacitive probe corresponding to the FZP motion [(2*c*), zoomed 2(*d*)] and the combined signal corresponding to the effective sample versus FZP motion [2(*e*) zoomed 2(*f*)] of one axis during a typical point-by-point scan. The scan positions were following a Fermat spiral trajectory (Huang *et al.*, 2014 ▸) with an average spacing of 0.6 µm. The tracking interferometer used in our ptychographic tomography scanner follows the motion of the sample stage. In order to minimize the vertical motion of the interferometer, the positions are sorted in vertical slabs of 3 µm-width. Within these slabs, a heuristic method based on the traveling salesmen sorting strategy is applied to optimize the scan path by minimizing the average step size as visualized in Fig. 3 ▸.

As can be seen in Figs. 2 ▸(*a*) and 2(*b*), due to its large mobile mass, the sample stage performs a rather smooth motion to the next position, while the FZP stage shows a spiky behavior shown in Fig. 2 ▸(*c*). Note that the long-range piezo actuator (Physikinstrumente P-563) is still driven at full force to reach the target positions. Therefore, without position compensation provided by the fast FZP scanner, this motion would result in an overhead of around 40 ms for a step size of 0.6 µm. The FZP effectively moves ahead until the required position of the sample versus the FZP corresponds to the desired target. At this moment the acquisition can begin. During acquisition, the sample stage continues to move to the desired target position while the FZP counteracts the motion of the sample stage, keeping the effective sample versus FZP position constant as can be seen in Figs. 2 ▸(*e*) and 2(*f*). It is therefore possible to reach a standard deviation below 10 nm during exposure, even though both the sample and FZP are actively moving.

### 2.1. FZP control loop implementation   

From a control point of view, the target positions and measured sample positions are fed to a control loop generating the control signal for the sample stage. The remaining error signal is fed to a second control loop for the FZP position with respect to the capacitive signal. At the beginning of a step in a scan, the position error is large, leading to the fast and large motion of the FZP. Once the sample reaches the target position, the error becomes close to zero and the FZP scanner reaches a position close to its center such that half its travel range is available for the next step in either direction. An even more optimal method of control could be to look ahead and already partially move with the sample towards the next target position within the travel range of the FZP scanner. Therefore the available travel range for the next step of the FZP scanner could be doubled without increasing variation of the illumination wavefront or reaching bounds determined by the OSA. This control mode is not implemented yet, but foreseen in the future.

### 2.2. Preparation of FZP with integrated CS   

**Figure 4:**
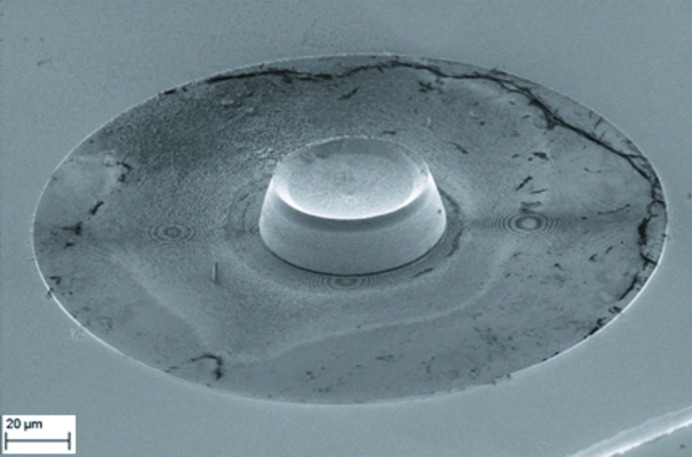
SEM micrograph (55° angle of view) of a gold Fresnel zone plate with an outermost zone width of 60 nm, height of 1.1 µm and a diameter of 200 µm, with an integrated central stop. The stop diameter is 50 µm with a height of 17.3 µm (center) and 20.6 µm (edges). PMMA resist residues that are mostly transparent to X-rays can be seen.

In general, the required motion by the FZP is very small, typically only a few hundred nanometres. To block the zeroth and higher-order light, a CS of 50 µm is used combined with an OSA of diameter 30 µm. To minimize the effect of the moving FZP on the illumination of the sample, it is crucial to guarantee synchronous movements of CS and FZP, otherwise the relative movement of the FZP around a static CS will become evident in the diffraction patterns and deteriorate the image reconstruction. In the present case, we have implemented the CS directly on the FZP. This has been done using a double-step e-beam lithography process. First, a gold zone plate with an outermost zone width of 60 nm and a height of 1.1 µm was created by electroplating in PMMA molds. The molds were realized by e-beam exposure at 100 keV (Vistec EBPG 5000plus, Raith GmbH) and further chemical development (Vila-Comamala *et al.*, 2010 ▸). Alignment markers positioned close to the zone plate were simultaneously written to locate the structures. Then, a second aligned exposure for the CSs was performed on a 20 µm-thick PMMA layer that was spin-coated on top of the gold structures. After development, gold CSs with diameter 50 µm were electroplated using a similar procedure, up to approximately 18 µm height (see Fig. 4 ▸). The heights of the central stops are not perfectly uniform and range from about 17.3 µm to 20.6 µm, possibly as a result of diffusion-limited mechanisms in the electroplating process. The PMMA layer was removed in warm acetone and the structures were critical-point dried to avoid mechanical collapse.

## 3. Evaluation   

The fast motion of the FZP can reduce the step response of our ptychotomographic setup (Holler *et al.*, 2012 ▸) by an order of magnitude to below 5 ms for a step length of 0.6 µm. Additionally, the high resonance frequency of the FZP scanner compared with the sample stage leads to a better overall stability of the setup because faster feedback can be applied to the FZP stage compensating for position vibrations in the sample stage. Table 1 ▸ shows the positioning times for various cases for a scan of a 15 µm × 10 µm region and 50 ms exposure time per position. The 419 scan positions were following the Fermat spiral trajectory (Huang *et al.*, 2014 ▸) with an average step of 0.6 µm and were either horizontally sorted in 3 µm-wide vertical slabs or optimally sorted using the traveling salesmen algorithm within the same slabs (see also Fig. 3 ▸). As can be seen, the acquisition rate can be increased from 8.9 Hz to 15 Hz by using the fast FZP scanner and the optimal sorting algorithm. While the total effective exposure time of the scan was 21 s, the total scan overhead was reduced from 26 s to 7 s, which also includes the overhead of setting up the scan in the instrument and initializing the X-ray detector.

**Figure 5:**
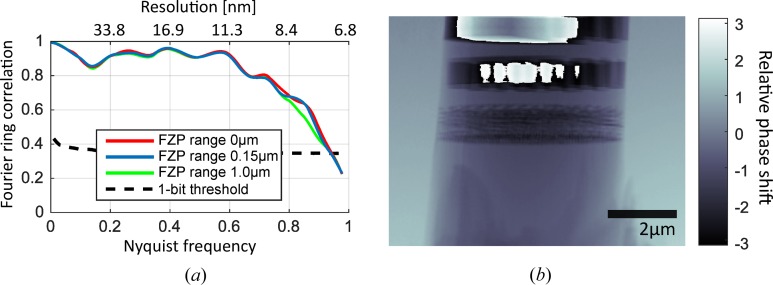
(*a*) Comparison of three Fourier shell correlation curves for different step sizes of the fast FZP scanner. (*b*) Example of the phase reconstruction of the test sample used.

In order to demonstrate that imaging quality does not significantly suffer by this approach, we imaged projections of an integrated circuit as shown in Fig. 5 ▸. The field of view was 9 µm × 7 µm, the exposure per position was 0.1 s and the step size was 0.4 µm. The total time per scan can be reduced from 64 s to 44 s, including 2 s for scan initialization. Therefore, the positioning overhead was reduced from 55% down to 5% by using the FZP scanner and optimal sorting within the slabs. Two identical projections have been acquired for both cases, reconstructed by the LSQ-MLc method (Odstrčil, Menzel *et al.*, 2018 ▸), and the resolution has been evaluated as the intersection of the Fourier shell correlation curve with the half-bit threshold (Van Heel & Schatz, 2005 ▸). Three scenarios were tested: no FZP movements, an FZP travel range limited to ±150 nm and an FZP travel range fully enabled. The non-moving FZP delivered slightly better reconstruction quality with a resolution of 7.4 nm compared with the full FZP travel range leading to a resolution of 7.5 nm. However, the differences in the estimated resolution are below 2%, and thus, for the targeted resolution, rather negligible.

Another approach for fast data acquisition is a continuous motion of the sample during exposure, the so-called fly scan method (Deng *et al.*, 2017 ▸; Pelz *et al.*, 2014 ▸; Odstrčil, Holler *et al.*, 2018 ▸), which is often used as a solution of scanning overhead for nanobeam ptychography. However, motion of the sample with respect to the illumination probe during sample exposure leads to a smearing and lower visibility of the speckles in the measured diffraction patterns. These smearing effects can be partially accounted for by the ptychographic reconstruction algorithm combined with a deconvolution method. Nevertheless, fly scan methods can cause up to a 100-fold increase in computational requirements compared with common step-scan methods and an additionally higher imaging dose is often needed to reach comparable quality with the common step-scan method as discussed in the work by Odstrčil, Holler *et al.* (2018 ▸).

**Figure 6:**
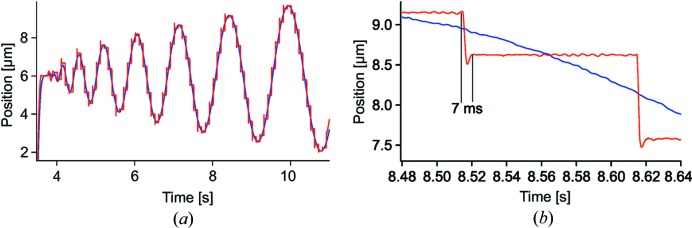
(*a*) Position evolution of the sample stage (blue) in one direction during a continuous spiral scan showing motion with practically constant velocity. (*b*) The FZP scanner is used to stabilize the effective position of the sample versus FZP (red) during exposure with a positioning overhead below 7 ms per 0.7 µm step as shown in the detailed evolution.

The fast FZP scanner can improve the situation and keep the effective position of sample versus FZP constant during exposure. An example is shown in Fig. 6 ▸, where the sample has been following an Archimedean spiral trajectory with 0.5 µm radial spacing, an average step length of 0.7 µm and exposure time 100 ms. Our hybrid scanning approach allows for a smooth, continuous motion of the sample stage at almost constant velocity. Note that Fig. 6 ▸ shows movement only along one axis. After the motion is triggered, the FZP scanner begins to ‘stabilize’ the relative position of FZP versus sample with respect to the newly provided target position which, in the present case, takes about 7 ms for a 0.7 µm step length. Then the position stability is maintained and a standard deviation of 6.4 nm is reached during the exposure. The ability and time to counteract the continuous motion depend on the scan parameters, since the travel of the sample during exposure has to stay within the travel range of the FZP scanner.

## 4. Conclusions and outlook   

We have developed a new approach for scanning X-ray microscopy by combining a slow sample stage possessing a long travel range with a fast but small range positioner for the beam-defining optics. The combined motion can significantly decrease the positioning overhead while preserving position accuracy and constant illumination as required by ptychographic reconstruction algorithms. We believe that our approach can provide an alternative to the currently used ptychographic fly scan methods without the need for increased computational demands, radiation dose, amount of acquired data or frame-rate of the available X-ray detectors.

## Figures and Tables

**Table 1:** Comparison of acquisition rates for various settings of the FZP scanner and position sorting for an average scan step of 0.6 µm and 50 ms exposure time

FZP scanner	Optimal sorting	Total time of scan (s)	Acquisition rate (Hz)
Off	Off	47	8.9
Off	On	45	9.3
On	Off	33	12.7
On	On	28	15.0
